# Determinants of Engagement in Leisure-Time Physical Activity and Club Sports among University Students: A Large-Scale Study

**DOI:** 10.3390/jfmk9030151

**Published:** 2024-08-29

**Authors:** Mohamad Motevalli, Clemens Drenowatz, Derrick R. Tanous, Gerold Wirnitzer, Werner Kirschner, Gerhard Ruedl, Katharina C. Wirnitzer

**Affiliations:** 1 Department of Sport Science, Universität Innsbruck, 6020 Innsbruck, Austria; 2 Department of Secondary Education, University College of Teacher Education Tyrol, 6010 Innsbruck, Austria; 3 Division of Sport, Physical Activity and Health, University of Teacher Education Upper Austria, 4020 Linz, Austria; 4 adventureV & change2V, 6135 Stans, Austria; 5 Research Center Medical Humanities, Universität Innsbruck, 6020 Innsbruck, Austria; 6 Department of Pediatric Oncology and Hematology, Charité—Universitätsmedizin Berlin, 10117 Berlin, Germany

**Keywords:** lifestyle, exercise, motivation, young adults, optimal health, public health

## Abstract

Various socio-demographic, environmental, and lifestyle-related factors have been reported to be associated with physical activity (PA) habits. However, there exist insufficient data comparing different forms of engagement in PA, sports, and exercise. This study aimed to investigate potential factors associated with the engagement in leisure-time PA (LPA) and club sports (CSs) in a large sample of college/university students. This Austria-wide study followed a cross-sectional design with a final sample of 4508 participants (mean age: 24.9 years; 65.9% female) from 52 Austrian colleges/universities. A standardized survey was used to collect data on demographics, anthropometric characteristics, and a wide range of health-related lifestyle factors, including patterns of PA and underlying motivations for PA engagement as well as details of dietary habits, sleep routines, smoking, and alcohol consumption. Descriptive statistics, chi-squared, logistic regression, and MANCOVA were used for data analysis. Across the entire sample, 85.7% of participants reported regular participation in LPA, including 22.5% who were active members of sports clubs. Of the 36 potential motives listed in the questionnaire, “maintaining physical health”, “feeling good”, and “refreshing the mind” were the most commonly reported factors motivating students to engage in either LPA or CSs. Ten socio-demographic, dietary, or lifestyle factors were identified as predictors of CSs participation (*p* < 0.01), whereas only two variables (specifically sleep-related factors) were identified as predictors of LPA participation (*p* < 0.001). These findings emphasize the importance of considering the type of PA and sport participation and the associated determinants when designing tailored strategies to promote an active lifestyle.

## 1. Introduction

An active lifestyle is associated with better quality of life and plays a crucial role in preventing and managing various chronic diseases, including obesity, cardiometabolic disorders, and psychosocial problems [1,2]. Recent trends highlight a widespread prevalence of unhealthy lifestyle choices, particularly sedentariness [3], and there is compelling evidence suggesting that participation in regular physical activity (PA), sports, and exercise can be an independent or complementary form of “medicine” for addressing various health conditions, including obesity [4,5,6].

Nowadays, maintaining sufficient PA levels has become more challenging due to the predominantly sedentary nature of daily routines. Data show that approximately 31% of adults worldwide have sedentary lifestyles, with prevalences varying from 17% in Southeast Asia to around 43% in America and the Eastern Mediterranean [7]. Results from other large-scale studies show that Austrian adults rank among the most physically active individuals in Europe [8]; however, PA levels have generally decreased among Austrian adult populations over the five-year period from 2014 to 2019 [9].

There are two prevalent modes of engagement in PA, including leisure-time PA and club sports participation. Leisure-time PA, which is often self-directed and less structured, takes place during free hours and is primarily based on individual preferences [10]. In contrast, club sports participation involves organized exercise and sport activities, typically involving scheduled practices and often including competitions [11]. These two modes of PA engagement have received significant attention in recent years because of their distinct characteristics and potential impact on physical, mental, and social well-being [12,13], which may vary by sex and age [11,14,15]. While leisure-time PA is reported to have a stronger impact on improving the quality of life and mental well-being [13,16,17], research indicates that sport club participation is associated with higher physical fitness, as well as enhanced skill development, along with providing competitive experiences [11,18].

Research shows that numerous lifestyle-related, sociodemographic, and environmental factors may influence an individual’s engagement in PA, sports, and exercise [19,20,21]. The integrated and interconnected nature of lifestyle behaviors highlights that PA patterns are linked to other lifestyle factors, such as dietary and sleeping habits, as well as stress, relationships, and substance abuse [22,23]. In addition, socioeconomic status and sociodemographic characteristics such as sex and age, as well as occupational or family circumstances, may influence patterns of PA and sport participation [24,25]. In light of these considerations, it is worth emphasizing that self-determined, autonomous motivation and intrinsic goals, such as enjoyment, improving health, reducing mental stress, managing chronic health conditions, fostering social connections, and skill development, play a crucial role in an individual’s decision to engage and persist in PA and sport programs [26,27,28].

Emerging (or young) adulthood is an important life stage, spanning from the late teenage years to the mid-twenties, during which individuals experience substantial developmental, psychological, and social changes [29]. It is crucial to emphasize that university life, which often coincides with emerging adulthood, is independently associated with psychological stressors, potentially resulting in additional adverse health consequences [30,31]. To date, there have been few studies investigating internal or external factors associated with PA or sport engagement in college or university students [31,32,33,34]. Data show that sedentary habits are prevalent among college/university students [32], with nearly half of them not engaging in PA and sport programs [33,34]. While these studies provide an initial insight into the factors influencing the rate of engagement, they are constrained by limitations such as small sample sizes and/or a lack of considering different types of PA engagement. The aim of this study was to investigate factors associated with engagement in leisure-time PA as well as club sports in a large group of Austrian college/university students. The ultimate goal is to provide a more comprehensive understanding of the factors influencing PA and sport engagement in college/university students, which can contribute to the development of tailored interventions and policies to promote a healthy and active lifestyle.

## 2. Materials and Methods

### 2.1. Study Design and Participants

The present study is a part of an Austrian study that examines health behaviors and their respective correlates in tertiary educational settings. This study follows a cross-sectional design with a multidisciplinary approach. This study targeted all college and university students enrolled in Austrian educational institutions. Students from 52 out of the 102 Austrian colleges and universities took part in the present study, which is supported by the Federal Ministry of Education, Science, and Research of Austria (BMBWF). The study protocol received approval from the ethics board of the respective tertiary educational entities. The study protocol was approved by the University of Innsbruck’s Board for Ethical Questions in Science (Certificate of good standing, 22/2021; 6 April 2021) and the Rectorate of the University College of Teacher Education Tyrol (PHT-HSa-17-Z1.8-5n_4927; 22 March 2021).

After receiving information about the objectives and procedure of this study, participants provided written informed consent prior to starting the survey. Participation was entirely voluntary and confidential, and students retained the option to withdraw their participation at any point without the necessity of providing a rationale. Participants accessed the online questionnaire through an encrypted interface via a web link. The survey was administered through LimeSurvey (version 3.25.15, LimeSurvey GmbH, Hamburg, Germany), accessible in both German and English.

### 2.2. Measures and Variables

The survey’s development process was based on a thorough examination of the pertinent literature, encompassing validated questionnaires and substantial scientific research, with all relevant sources readily accessible in the study protocol [35]. The standardized questionnaire encompassed a diverse array of inquiries spanning various facets of participant demographics (including age, sex, marital status, nationality, living environment, and federal state of residence), personal biometrics (body weight and height), and an extensive array of health-related lifestyle factors, including dietary habits, sleep routines, smoking and alcohol consumption, and patterns of PA. Additionally, participants answered questions regarding their underlying motivations for engaging in recreational PA and sports. Participants were presented with a variety of question types, including single- and multiple-choice items, as well as the opportunity to express their preferences by selecting and rating from a range of options. Additionally, the online questionnaire incorporated several control questions designed to detect inconsistencies in responses, thereby enhancing the overall reliability of the collected data.

To determine PA engagement of the participants, they were initially asked about their regular involvement in PA. Subsequently, those indicating regular participation in PA were further questioned to determine whether their engagement took the form of sports club activities or not. Consequently, participants were categorized based on their responses to these two distinct questions, classifying them as either engaged in leisure time PA or not and involved in club sports or not.

**Figure 1:**
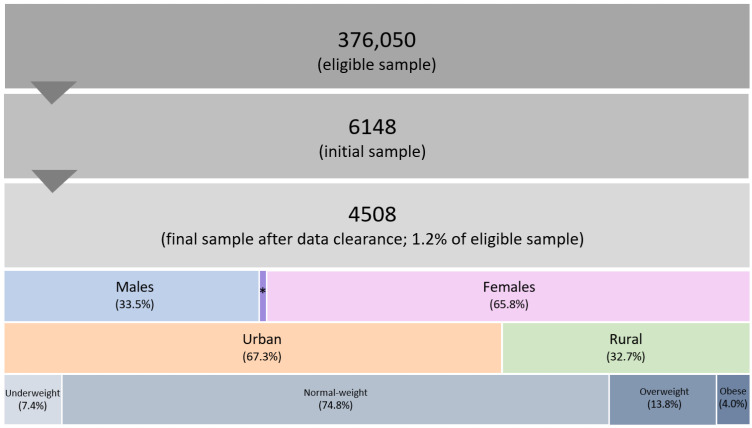
Sample size and distribution of participants based on sociodemographic characteristics. * Diverse population, representing 0.7% of the final sample size.

BMI was determined through self-reported body weight and height measurements, with the WHO-defined BMI cut-off points utilized to classify participants into specific BMI subgroups, including underweight (<18.5 kg/m^2^), normal-weight (18.5–24.9 kg/m^2^), overweight (25.0–29.9 kg/m^2^), and obese (>30.0 kg/m^2^) [36]. Figure 1 displays the sample size and categorization of participants based on their sociodemographic factors.

### 2.3. Statistical Analysis

All statistical analyses were conducted using SPSS (version 26.0, IBM SPSS Statistics, Chicago, IL, USA). Exploratory analysis was carried out using descriptive statistics, and the data are presented as mean with standard deviation (±SD) for continuous data and as prevalence (percentage) for nominal data. Multivariate analysis of variance (MANOVA) was used to examine sex-based differences in anthropometric characteristics and age. Chi-square tests were conducted to examine sex differences in the prevalence of PA/sport participation. Logistic regression analysis, with a 95% confidence interval (95%-CI), was used to identify significant predictors of participation in either leisure-time PA or sports club engagement. The statistical significance level was set at *p* ≤ 0.05.

## 3. Results

A total of 4508 students from all nine federal states of Austria, representing 1.2% of the Austrian student population, provided valid and complete data. Across the entire sample, 85.7% indicated regular participation in leisure-time PA, and 22.5% were actively involved in sports clubs. While there was no significant sex difference in leisure time PA (*p* > 0.05), participation in club sports was significantly more common among male participants (*p* < 0.01) (Table 1).

Among the 36 potential motives listed in the questionnaire, the most frequently mentioned motives for participating in leisure-time PA were “maintaining physical health” (12.9%), “feeling good” (11.3%), and “refreshing the mind” (10.0%). These three motives also ranked highest for participants engaged in club sports, albeit with slight variations in their prevalence (Table 2). “Maintaining body shape” (5.1%) emerged as the next commonly mentioned motive for leisure-time PA participants, while the next most commonly mentioned motive for engaging in club sports was “enjoying sports engagement” (6.0%).

Participants who regularly participated in leisure-time PA had a significantly lower BMI compared to those who reported no engagement in PA (*p* < 0.01); however, this difference in BMI was not observed with club sports participation (*p* > 0.05). Participation in leisure-time PA or club sports showed no significant association with age (*p* > 0.05). A significant trend towards better health ratings was observed among participants who engaged in either leisure-time PA or club sports (*p* for trend < 0.01) (Table 3). After adjusting for sex, all findings remained consistent.

The results of the logistic regression analysis (Table 4) revealed that among the various study variables, certain clusters (including “sociodemographic variables”, “dietary/lifestyle variables”, and “sleep-related variables”) and individual factors (including “being Austrian”, “living in rural area”, “daily consumption of fruits”, “consuming fluids > 2 L/d”, “avoiding smoking”, “avoiding alcohol”, and “rating sleep as the 1st health factor”) are significant correlates for participation in club sports (*p* < 0.001–*p* = 0.008). Furthermore, the category of “sleep-related factors” as a whole and the “feeling well-rested after sleep” variable emerged as notable correlates for participation in leisure-time PA (*p* < 0.001). The factors “avoiding alcohol” and “avoiding smoking” did not emerge as significant correlates for engagement in leisure-time PA (*p* > 0.05).

## 4. Discussion

The present study, involving a sample of 4508 Austrian college and university students, aimed to investigate potential factors associated with participation in leisure-time PA and club sports. Key findings include the following: (i) the majority of participants (85.7%) reported regular leisure time participation and 22.5% were active members of sports clubs; (ii) the most common motivations for participating in both leisure-time PA and club sports were “maintaining physical health”, “feeling good”, and “refreshing the mind”; (iii) participants with regular engagement in leisure-time PA had a lower BMI compared to those who reported no engagement in PA (*p* < 0.01); (iv) participants engaging in leisure-time PA or club sports had a higher prevalence of being in the normal-weight category (*p* < 0.01); (v) no significant age or sex differences were found in the prevalence of engagement in leisure-time PA (*p* > 0.05), while participation in club sports was significantly more common among male participants (*p* < 0.01); (vi) across various socio-demographic and behavioral variables, ten were significantly associated with club sports participation (*p* < 0.01), while only two variables were significantly associated with leisure-time PA participation (*p* < 0.001).

College and university students are at a crucial stage of life where they can establish sustainable and lifelong health habits [31,37]. Data from large-scale investigations indicate that 41–72% of college/university students fail to meet the minimum guidelines for health-promoting PA [38,39,40]. In the present study, however, the majority of the students reported regularly participating in leisure-time PA. This finding aligns with WHO reports indicating that approximately 75% of adults in Austria adhere to recommended weekly PA levels, locating them among the most physically active people in Europe [8]. While nationality may emerge as a potentially influential factor accounting for the heightened engagement of the Austrian population in PA, it is crucial to acknowledge that the inconsistency between our findings and the available data may be attributed to methodological variations, including the PA assessment approach in the present study that did not offer a precise identification of the proportion of participants who met the minimum PA guidelines. In the present study, 22.5% of physically active students (accounting for 19.3% of total participants) reported being actively involved in a sports club, with male students displaying a greater tendency for involvement in club sport activities compared to their female peers. The overall participation rate in club sports closely matches the findings of a study conducted in Slovenian university students [41]. However, it is crucial to acknowledge that there are inconsistencies regarding sex-based differences, as two previous studies report that male and female university students exhibited equal levels of engagement in club sport activities [41,42]. This sex-based difference was not observed in the context of leisure-time PA engagement among students in the present study, which is consistent with the findings from another study conducted among American university students [43].

Various external factors can influence students’ engagement in club sports. Notably, college/university students commonly face time management challenges due to the frequent changes in their study schedules, leading to subsequent disruptions in their daily routines and making it difficult for them to maintain an organized PA/sport program [40,44]. Moreover, research indicates that financial constraints are often the primary barrier for university students’ participation in club sports [45], leading them to seek cost-free alternatives of PA. Data show that factors like the presence of sports facilities or natural surroundings like mountains or hiking paths can affect students’ setting-related choices to pursue PA [46,47]. A lack of necessary skills for joining a sports club can potentially hinder novice individuals from gaining access, and this preliminary limitation seems to be a significant obstacle to their participation in club sport activities.

Apart from external factors, a range of internal factors and motivations can significantly impact an individual’s engagement in PA/sports [26,27,28]. The present study has discovered that the top three motivations for engaging in both leisure-time PA and club sports among young adults are similar, with “maintaining physical health” being the most frequently cited reason. Although it is unclear whether leisure-time PA or club sports participation should take precedence in terms of health benefits, research shows that both forms can substantially enhance overall health and well-being [48,49]. Consistently, evidence indicates that people participating in recreational sports, regardless of the type, were mainly motivated by health principles [11]. Results from another study, however, indicate that adults exercising in natural settings are primarily motivated by convenient accessibility in terms of time, location, cost, and the ability to practice at their own pace, while gym members or participants in organized sports emphasized physical health and sociability as their primary motivators [11,50], especially under the supervision and guidance of a fitness or health coach/instructor [51]. According to the available data, differences in motives for participating in PA/sports may be linked to demographic variables, including sex [26,52,53]. In this regard, data suggest that females typically find greater enjoyment in recreational PA activities characterized by inclusiveness, while males gravitate towards activities with a competitive nature [54]. In a study examining the impact of sociodemographic factors on exercise/sport motivation among college students, it was found that males were primarily motivated by intrinsic factors (including strength, competition, and challenge), while females were more influenced by extrinsic factors (such as weight management and appearance concerns) [53]. In the present study, however, no substantial differences in motivation-related factors were observed among different categories of sex, living environment, and nationality. The contrast between these findings and the available data can be explained by the influence of various factors, including cultural context and individual backgrounds, which have the potential to diminish the conventional sex-based differences observed in previous research.

In the present study, the prevalence of overweight/obesity was 17.8%, which is remarkably below both the average for Austrian young adults (29.4%) [9] and the finding from another study involving American university students (28.4%) [43]. Considering the strong relationship between physical inactivity and obesity rates [55], the low prevalence of overweight/obesity in the present study may be justified by the high prevalence of their engagement in PA/sport. Further, the findings in the WHO European Regional Obesity Report 2022 show that Austrian adults have one of the lowest rates of age-standardized overweight/obesity in Europe [56]. In the present study, students who regularly engaged in leisure-time PA had a lower BMI and were less likely to have excess body weight compared to those with no PA/sports involvement. Increased PA has been shown to result in a more dynamic metabolism and better regulation of energy intake [57], ultimately enhancing the body’s ability to reach or maintain a healthy BMI range [58,59]. However, studies conducted on university students have yielded contradictory findings regarding the BMI values. A study in Korean students indicated no difference in BMI values between physically active and non-active university students [60], and another study in American university students reported no distinction in leisure-time PA engagement rates between those with and without excess body weight [43]. These contradictory findings can be attributed to methodological factors, particularly variations in study methodologies. It should also be noted that BMI has limitations in distinguishing between fat mass and fat-free mass, which is particularly critical for participants in certain sports who may have greater muscle mass due to the nature of their sport. However, in the present study, the average BMI of the participants in the two study groups was almost the same (22.4 and 22.5 kg/m^2^).

In this study, avoiding alcohol and avoiding smoking did not emerge as significant correlates for leisure-time PA engagement, while both variables were found to be significant indicators for engagement in club sports. Findings from a large-scale investigation among university students indicate a lack of significant associations between smoking or alcohol intake and PA engagement [60], which align with data from the present study, specifically with regard to leisure-time PA. The adverse effects of substance abuse on the academic performance of university students, nevertheless, have been well documented [61]. However, evidence reveals a high prevalence of smoking and alcohol consumption (ranging from 40% to 70%) in college/university settings [62,63,64], with a higher incidence among male students [63,65]. In the present study, while several sociodemographic, dietary, lifestyle, and sleep-related factors were observed to be significantly associated with the rate of club sports participation, only the latter, specifically the variable “feeling well-rested after sleep”, emerged as a predictor for engagement in leisure-time PA. These data suggest that lifestyle choices and factors related to PA engagement can vary significantly based on the context of the activity, and particularly, students who engage in club sports may have a distinct set of motivations and lifestyle considerations compared to other students. The significance of sleep as a predictor for participation in both leisure-time PA and club sports highlights the substantial role of getting sufficient and quality sleep in reloading energy levels and motivating individuals to be physically active [66,67], suggesting that the student’s capacity to recover and feel recharged after sleep is important in determining their readiness to participate in any form of PA.

This study had some limitations worth highlighting. This study’s cross-sectional design, which relies on self-reported data, could result in socially desirable responses in the form of under-reporting or over-reporting. In addition, only regular PA/sport participation was examined, which makes it impossible to determine the proportion of students meeting PA recommendations. Research, nevertheless, suggests that single-item questions are considered a reliable and commonly used method in the social and behavioral sciences, offering the benefit of reducing the problems associated with lengthy surveys [68,69]. Despite these limitations, this study possesses several strengths. The large sample size along with a nationwide distribution enhances the representativeness of the findings. Additionally, this study represents one of the initial endeavors to explore a diverse range of factors associated with participation in leisure-time PA and sports clubs, as two popular domains for engaging in physical exercise.

The present study provides insight into the factors that influence leisure-time PA and club sports participation among university students. Understanding these determinants can inform targeted interventions and strategies to promote active lifestyles. By considering individual motivations, sociodemographic factors, and lifestyle choices, effective programs can be designed to promote sustainable PA in educational settings. However, future research can contribute to a more comprehensive understanding of PA behaviors. Using longitudinal research approaches and following participants over time is critical for a deeper exploration of changes in PA behaviors, motivations, and associated factors. In addition, comparing PA behaviors and motivations across demographic groups (e.g., socioeconomic status, academic disciplines) may be needed to identify disparities and inform more targeted approaches. It is also important for future studies to incorporate more advanced variables beyond BMI (such as measuring fat mass, lean mass, and waist-to-hip ratio) that provide a more accurate description of body composition and its association with the type of PA engagement.

## 5. Conclusions

The present study showed that the majority of Austrian university students regularly engage in PA during their leisure time, while about one out of five students participate in club sport activities. The findings indicate that motivational factors for participation in either leisure time PA or club sports are similar in this group. In addition, participation in each of these two forms of PA is influenced by a range of internal and external factors, suggesting that the type of engagement in PA/sport is a crucial aspect of a healthy lifestyle. These findings provide viable information on influences on PA and club sport engagement in tertiary educational settings. Understanding key factors that influence participation in PA and sports can help health and educational administrations in developing tailored strategies to promote a culture of an active lifestyle among university students as well as young adults.

## Figures and Tables

**Table 1:** Anthropometric characteristics and PA/sports participation by sex. Values are presented as mean ± SD or prevalence (%).

	Total (n = 4508)	Females (n = 2969)	Males (n = 1509)	Diverse (n = 30)
Age (years) ^1^	24.9 ± 6.3	24.4 ± 6.1	25.7 ± 6.7	27.6 ± 8.2
Height (cm) ^1,3^	171.9 ± 9.2	167.2 ± 6.2	181.2 ± 6.8	168.3 ± 9.7
Weight (kg) ^1,2,3^	66.9 ± 14.1	61.6 ± 11.0	77.3 ± 13.6	68.3 ± 16.5
BMI (kg/m^2^) ^1,2^	22.5 ± 3.7	22.0 ± 3.6	23.5 ± 3.7	24.0 ± 5.0
Leisure-time PA (%)	85.7	86.6	84.0	80.0
Club Sports (%) ^1^	22.5	20.4	26.6	20.0

^1^ Significant difference between female and male participants (*p* < 0.01). ^2^ Significant difference between female and diverse participants (*p* < 0.01). ^3^ Significant difference between male and diverse participants (*p* < 0.01).

**Table 2:** Top three motives for participation in leisure-time PA (n = 3863) and club sports (n = 1014) represented by sex, living environment, and nationality.

		Total	Male	Female	Urban	Rural	Austrian	Int.
Maintaining Physical Health	Leisure-time PA	12.9%	12.6%	13.1%	12.6%	13.5%	13.2%	11.9%
	Club Sports	11.7%	12.1%	11.5%	11.3%	12.4%	11.9%	10.5%
Feeling Good	Leisure-time PA	11.3%	10.2%	11.8%	11.8%	10.4%	10.9%	12.9%
	Club Sports	10.0%	9.8%	10.0%	9.9%	10.5%	9.8%	11.0%
Refreshing the Mind	Leisure-time PA	10.0%	9.3%	10.2%	9.8%	9.9%	9.7%	10.4%
	Club Sports	10.0%	9.1%	10.7%	10.1%	9.9%	9.4%	13.6%

Int.—international; PA—physical activity.

**Table 3:** Differences in anthropometric characteristics and self-reported health rating by PA/sport participation.

		Leisure-time PA		Club Sports	
		Yes	No	Yes	No
Age (years)		24.8 ± 6.3	24.9 ± 6.7	24.4 ± 6.2	25.0 ± 6.4
Height (cm) ^2^		171.9 ± 9.2	172.0 ± 9.3	173.1 ± 9.6	171.5 ± 9.1
Weight (kg) ^1^		66.4 ± 13.1	69.8 ± 18.5	67.7 ± 12.9	66.7 ± 14.4
BMI (kg/m^2^) ^1^		22.4 ± 3.3	23.5 ± 5.3	22.5 ± 3.1	22.6 ± 3.9
BMI Classes	Underweight ^1,2^	6.9%	9.9%	4.8%	8.1%
	Normal-weight ^1,2^	77.1%	61.1%	80.1%	73.3%
	Overweight ^1,2^	12.9%	19.4%	11.9%	14.4%
	Obese ^1^	3.1%	9.6%	3.2%	4.2%
Health Rating ^3^	Excellent	16.6%	4.0%	20.5%	13.1%
	Very Good	46.1%	27.9%	46.4%	42.6%
	Good	31.2%	48.5%	27.8%	35.4%
	Moderate	5.7%	17.7%	5.0%	8.2%
	Bad	0.4%	1.9%	0.3%	0.7%

^1^ Significant difference by leisure-time PA participation (*p* < 0.01). ^2^ Significant difference by club sports participation (*p* ≤ 0.01). ^3^ Significant tendency toward a better health rating with leisure-time PA and club sports (*p* for trend < 0.01). Note: All results remain unchanged after adjusting for sex (using MANCOVA).

**Table 4:** Results of the final logistic regression analysis showing the significant predictors of engagement in leisure-time PA and club sports.

	Type of Engagement	Exp(B)	CI	*p*-Value
Sociodemographic variables	Club sports	0.214	---	*p* < 0.001
Being Austrian	Club sports	1.345	1.106–1.636	*p* = 0.003
Living in rural area	Club sports	1.329	1.147–1.540	*p* < 0.001
Lifestyle/Dietary variables	Club sports	0.164	---	*p* < 0.001
Daily consumption of fruits	Club sports	1.229	1.055–1.432	*p* = 0.008
Consuming fluids > 2 L/d	Club sports	1.507	1.307–1.737	*p* < 0.001
Avoiding smoking	Club sports	1.577	1.235–2.014	*p* < 0.001
Avoiding alcohol	Club sports	0.830	0.707–0.975	*p* = 0.023
Sleep Variables	Leisure-time PA	6.235	---	*p* < 0.001
	Club sports	0.181	---	*p* < 0.001
Feeling well-rested after sleep	Leisure-time PA	2.611	2.119–3.102	*p* < 0.001
Rating sleep as the 1st health factor	Club sports	1.278	1.102–1.483	*p* = 0.001

Exp(B)—exponential value of B (the odds ratio), representing how the odds of the event happening multiply when the independent variable changes by one unit; CI—confidence interval; PA—physical activity.

## Data Availability

Data is contained within the article.
